# Leveraging retooled clinical research infrastructure for Clinical Research Management System implementation at a large Academic Medical Center

**DOI:** 10.1017/cts.2023.550

**Published:** 2023-05-18

**Authors:** Catherine G. Mullen, Jessica Y. Houlihan, Marissa Stroo, Christine E. Deeter, Stephanie A. Freel, Angela M. Padget, Denise C. Snyder

**Affiliations:** 1 Duke Office of Clinical Research, Duke University School of Medicine, Durham, NC, USA; 2 Clinical and Translational Science Institute, Duke University, Durham, NC, USA

**Keywords:** Clinical trial management system, academic medical center, clinical research finance, clinical research operations, clinical trial efficiency

## Abstract

Quality clinical research is essential for health care progress and is the mission of academic health centers. Yet ensuring quality depends on an institution’s ability to measure, control, and respond to metrics of trial performance. Uninformed clinical research provides little benefit to health care, drains institutional resources, and may waste participants' time and commitment. Opportunities for ensuring high-quality research are multifactorial, including training, evaluation, and retention of research workforces; operational efficiencies; and standardizing policies and procedures. Duke University School of Medicine has committed to improving the quality and informativeness of our clinical research enterprise through investments in infrastructure with significant focus on optimizing research management system integration as a foundational element for quality management. To address prior technology limitations, Duke has optimized Advarra’s OnCore for this purpose by seamlessly integrating with the IRB system, electronic health record, and general ledger. Our goal was to create a standardized clinical research experience to manage research from inception to closeout. Key drivers of implementation include transparency of research process data and generating metrics aligned with institutional goals. Since implementation, Duke has leveraged OnCore data to measure, track, and report metrics resulting in improvements in clinical research conduct and quality.

## Introduction

Clinical research remains a critical mission for academic medical centers (AMCs) [1]. In 1999, a Clinical Research Task Force from the Association of Academic Medical Colleges (AAMC) called on AMCs to revolutionize clinical research and facilitate best practices in health care [2]. This Task Force advised institutions to strengthen clinical research infrastructure to support science, care delivery, and financial acumen. Furthermore, leadership across AMCs expressed concerns that the clinical research workforce and infrastructure needed to be strengthened to keep up with advances in basic science research [3]. While AMCs have been committed to meeting these objectives, major barriers, including a lack of trained researchers, integrated systems, standardized policies and procedures, competent staffing, and appropriate resources persist [4,5]. In particular, fragmented infrastructure, coupled with inexperienced staff and high turnover, was adversely affecting the quality and value of clinical research [6,7].

Even as a highly regarded AMC, Duke’s clinical research ecosystem had struggled for years with highly decentralized research activity. This limited our ability to identify, measure, and enhance study success. With little central support and oversight, problems loomed, ranging across several areas including billing compliance and data integrity. To address this challenging set of circumstances and heed the charge of the AAMC, Duke University School of Medicine committed resources for three major initiatives: (1) restructuring central oversight and support; (2) strengthening the clinical research workforce; and (3) implementing a central clinical research management system (CRMS).

In 2012, Duke restructured the central research support office and created the Duke Office of Clinical Research (DOCR). In the structure created by DOCR, departments wishing to create a Clinical Research Unit (CRU) must submit a letter of intent detailing the therapeutic area, rationale for creating a new CRU and how the proposed CRU will fit into the existing structure, to the Vice Dean for Clinical Research for review and approval. All CRUs are governed by a charter outlining leadership, scope of research under its oversight, interactions with a faculty advisory board, governance, and financial plan. As described by Snyder *et al*. [4], the new DOCR team focused on implementing standard policies, procedures, and training to support study start-up, conduct, and closeout processes while also working with the existing departmentally based CRUs to strengthen the research oversight practices within each group. Following the successes of this early infrastructure work, we embarked on several initiatives to strengthen our clinical research workforce [4,8–10]. These Workforce Engagement & Resilience Initiatives created consolidated and standardized job classifications and career advancement pathways that paved the way for central tracking of hiring and attrition data, targeted onboarding training, and role-based access controls to clinical research systems [7].

While the centralized support structure and workforce reclassification initiatives resulted in numerous improvements, we still lacked the ability to efficiently access institution-level data about our research portfolio, which is key to prioritizing and evaluating improvement efforts [11]. Clinical research units existed in silos, each using different processes and systems to manage their portfolio with limited standardization or transparency. No single source existed to track clinical research project lifecycle statuses or progress. IRB continuing review data was updated only annually, and those data could not be easily extracted for reporting. Financial management of industry-funded trials was being managed on project-specific spreadsheets. There was no ability to efficiently report portfolio metrics at the CRU or institutional level. Requests for this type of data required tedious manual work to collect, standardize, and report fundamental data to stakeholders.

In 2018, Duke addressed these issues by implementing Advarra’s OnCore CRMS (Advarra, Madison, WI). Key objectives of this implementation were to foster data transparency and allow for the collection and assessment of metrics aligned with quality and institutional goals. OnCore, including OnCore Financials, was launched enterprise-wide, with integrations between Duke’s electronic health record, Epic (Epic Systems Corporation, Verona WI), the electronic IRB system, iRIS (Cayuse, Portland OR) and the general ledger, SAP (SAP, Walldorf, Baden-Württemberg Germany). “CRU” was used as a unifying variable in each system. The overall goal was to create a standardized experience to manage clinical research from protocol creation to project closeout. Since implementation, Duke has leveraged the data tracked in OnCore to measure and report metrics, resulting in improvements in clinical research quality and transparency, while identifying priorities for monitoring and improvements. These systems along with enhanced business infrastructure were critical to the institution’s ability to pivot during the COVID-19 pandemic. The commitment to boost resources and reinforce the institutional commitment to clinical research remains a priority for changing health care in our community.

## Methods

An enterprise-wide implementation of a CRMS is a complex task and involved years of preparation by a core leadership team, along with close collaboration with the research community [12]. Duke employed the following strategies to ensure that the implementation resulted in a widely adopted, well-understood, and richly populated solution that would provide valuable insight into the performance and quality of research in progress.

### Engage Research Community Stakeholders

Identifying and engaging members of the research community were critical for successful implementation. The community participated in the selection of the vendor system and provided input on operational workflows. We used a targeted approach for training, given OnCore’s wide-ranging capabilities and the multiple new workflows being introduced to our research community. OnCore access was based on functional role (for example, users who manage subject-level information have the Subject Management user role). We leveraged the job classification structure established in 2016 to target members of the workforce with the appropriate OnCore training and support, training 2,000 users via e-learning in less than 45 days. At go-live, 75 staff members from the research community supported the implementation as OnCore Champions. Staff members included department leadership, grants managers, and clinical research professionals across CRUs. Champions used toolkits developed by the central team to provide at-the-elbow assistance for front-line CRU staff. To increase engagement and adoption of the new system, monthly Champion meetings were held to discuss workflows and provide tools and topics for dissemination within their CRU.

### Focus on Study Start-up Workflow

The study start-up and approval process for clinical research is a complex process involving multiple stakeholders [13]. With the implementation of OnCore, we were excited to create a robust workflow to track study start-up progress and guide process improvement. The implementation of OnCore Financials created an opportunity to track the timeline for study budget creation and negotiations for industry-funded studies. In the new workflow, all new protocols are entered into iRIS and pushed over to OnCore via an interface. Studies are triaged by the DOCR Study Start-up team based on the needs of the protocol. Protocols that include activities within the health system are required to have an OnCore calendar, coverage analysis, and Epic build (order set or treatment plan). The Start-up team meets with the study team after reviewing their protocol and draft sponsor budget. At the meeting, the OnCore calendar, Epic orders (including labs and procedures) and billing designations are reviewed with the Principal Investigator (PI) and Study team, and feedback is collected. After the meeting, the OnCore Calendar is sent to the PI and Study team for approval. When approval is received, an Epic analyst builds the order set or treatment plan based on the OnCore Calendar. The draft Epic build is sent for validation by the PI and Study team and moved into Epic production once approved. The OnCore calendar along with billing designations are pushed over to Epic.

Each CRU has financial responsibilities. The finance team uses the coverage analysis and protocol to ensure all expenses are incorporated into the budget for negotiations with the Sponsor. Negotiated rates for milestones and invoiceables are entered in OnCore Financials once a contract has been agreed upon. Budget reviews and approvals are documented in OnCore. To receive Institutional Approval, a protocol must receive IRB approval, have a fully executed contract and approved budget in OnCore (if applicable), a complete Clinical Trial.gov registration (if applicable), an approved data storage plan and an approved quality monitoring plan. OnCore Task Lists are used to manage each step of this process. Task Lists track the role and individual responsible for each step in the process and send reminders to the individual to complete an outstanding task.

### Pilot OnCore Financials

Based on the complexity of implementing new budgeting and invoicing workflows for industry-funded protocols, OnCore Financials was piloted with a group of six CRUs varying in size and portfolio complexity. The pilot group developed workflows for budgeting (incorporating National Coverage Determination guidelines), invoicing, reconciliation, and payment. A real-time interface to the general ledger picks up finalized post-award invoices, which are then posted in the general ledger as revenue. Payments received and posted by central finance in the general ledger are sent to OnCore via a daily file transfer, and line-item reconciliation of all payments occurs in OnCore. Additional CRUs were onboarded to OnCore Financials quarterly in launch groups consisting of four to five CRUs over 24 months.

### Building an Accurate Dataset

Data transparency and metrics were key objectives of the OnCore implementation. While portfolio management was a focus of the OnCore implementation, optimal portfolio administration depends on complete and accurate data. To that end, we identified protocol metadata, lifecycle status, and participant enrollment and visit tracking as key areas for portfolio and protocol management. Policies and procedures were developed to define expected timeframe and required documentation in OnCore. The base of data entry in OnCore starts with completion of the Minimum Footprint, a standard OnCore report of protocol metadata fields, customizable by organization. We decided on eight required fields for studies in process prior to implementation. For new studies after implementation, the required fields, many populated by the OnCore iRIS interface, were expanded to 36 with additional fields added for Oncology studies (Table 1). These required fields make up the dataset to create customized reports (e.g. National Cancer Institute required reporting), produce metrics, and display in dashboards. To hold CRUs accountable for accurate and complete data, we added a minimum footprint completion goal on the CRU Scorecard, as described in greater detail below.

**Table 1.** Duke Minimum Footprint data fields for new studies

Accrual duration	Total accrual goal (Lower)	Oncology group**	Protocol target accrual
Accrual information applicable?	Total accrual goal (Upper)	Organizational unit*	Protocol type
Affiliate accrual goal	Exclude from web	Phase*	Rare disease
Age	Grant/Contract	Principal Investigator*	Short title*
Current protocol status	Institution	Precision trial **	Sponsor role
Current protocol status date	Investigator initiated protocols*	Precision trial classification**	Sponsor*
Data monitoring	IRB number*	Primary completion date	Study completion date
Data Table 4 report type**	Library*	Primary management group	Study coordinator*
Department	Management group	Principal sponsor	Title*
Disease/diagnosis group	NCI trial ID**	Program area**	Toxicity scheme**
Annual accrual goal	NCT number	Protocol number*	

* Data field populated via the OnCore iRIS interface.

** Additional fields required for studies in the Oncology library.

NCI trial ID-National Cancer Institute trial ID.

NCT number- National Clinical Trials number.

### Augmenting the CRU Scorecard

The CRU Scorecard was started in 2014 at the request of the Dean’s office to address areas that needed improvement across the research enterprise. The CRU Scorecard offers a balanced view of each CRU by reporting financial and nonfinancial metrics over time. Key areas include enrollment, CRU protocol review times, OnCore use (subject visit tracking, timely study closeout, summary accrual, minimum footprint), and review of OnCore Financials (industry budgets, review and signoff, timely invoicing). A target performance goal is assigned to each metric, and a stoplight system is used to assess and display performance over the review period. While some of these metrics change annually, others persist for years, allowing us to trend progress and monitor ongoing compliance and system adoption. Scorecard metrics are reported quarterly to CRU leadership and are scored annually. Today, OnCore provides data for most of the scorecard calculations. For the past few fiscal years, the data have shown improvements across units in enrollment, operational adoption, and financial attention.

## Results

The implementation of OnCore has allowed Duke to establish a baseline of more accurate and complete data across our entire clinical research portfolio. Improved data have allowed us to make data-driven decisions leveraging oversight policies, procedures, and enhanced analysis capabilities. Initial focus areas included CRU Scorecard metrics, enrollment and demographics, and study start-up timeline metrics.

### CRU Scorecard Metrics

Since the Minimum Footprint is the foundation for all OnCore reporting, it is a requirement for all studies. We have tracked compliance with the Minimum Footprint as a metric across all CRUs since fiscal year 2019 (FY19). Minimum Footprint data points are reviewed for accuracy by the CRU operational leaders. The metric is based on the number of studies marked as “Review Complete” in OnCore divided by the number of studies institutionally approved for the quarter. CRUs achieving > 95% receive a green light, 94–90% a yellow light, and < 90% a red light. In FY19, 18 CRUs received a green light and 3 received red lights. As in many of our metrics, we witnessed a decline in adherence during the COVID-19 pandemic as study teams faced operational and staffing challenges, resulting in 13 CRUs scoring a green light in FY20 and 14 in FY21. In FY22, we saw an improvement, with 18 CRUs receiving a green light, 1 receiving a yellow, and 2 receiving reds.

### Enrollment Pace and Demographics

**Figure 1.**
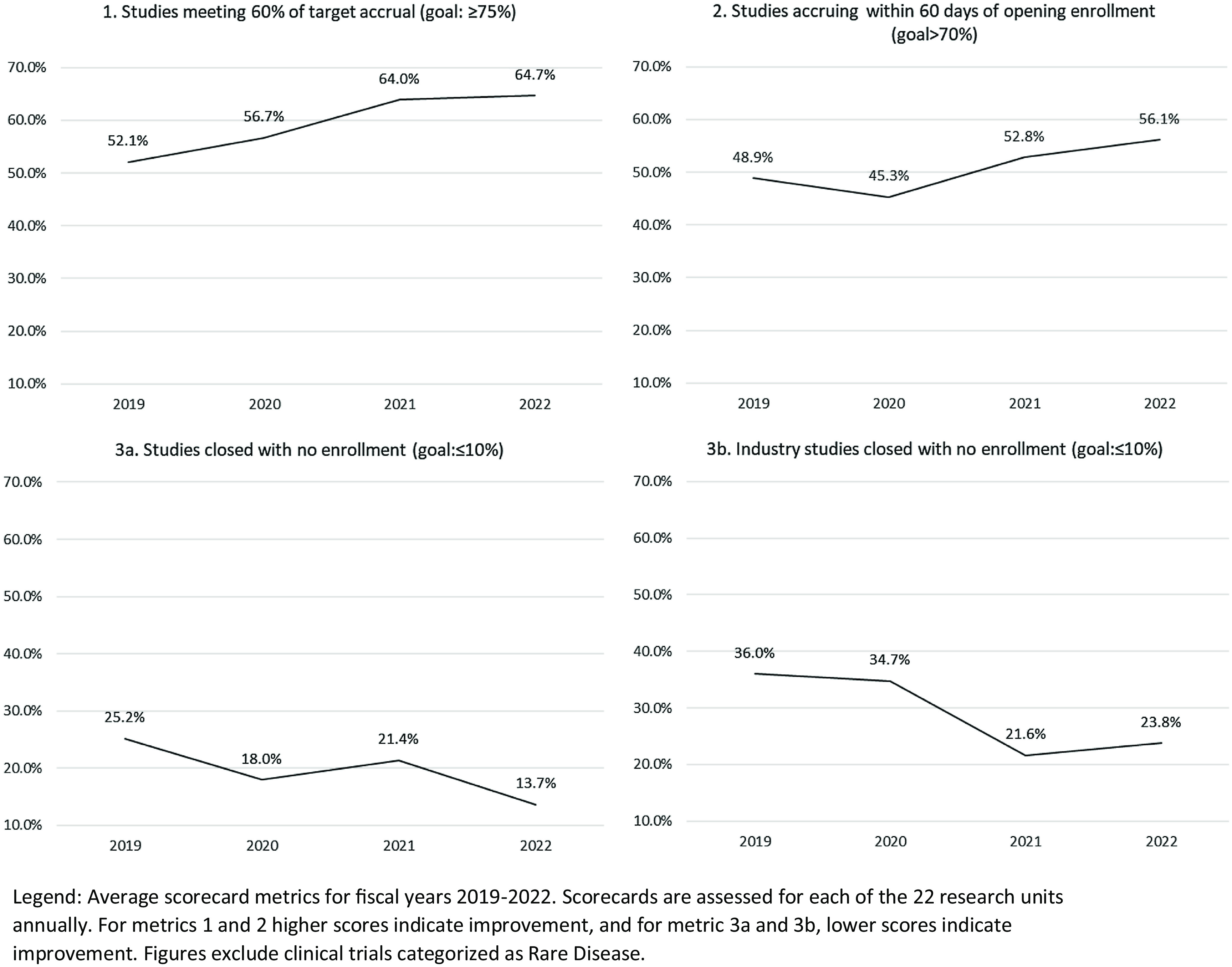
Average scores on enrollment study metrics for fiscal years 2019–2022.

Participants are registered in OnCore upon consent to a study. This allows for near real-time enrollment and demographic reporting. We have tracked three portfolio-level enrollment metrics since OnCore implementation excluding studies meeting the Rare Disease designation. These metrics are arbituary, set based on available data at implementation and established as stretch metrics to indicate progress. Favorable trends have been noted across all three metrics (Fig. 1). The percentage of studies meeting 60% of target accrual has increased from 52.1–64.7%. Studies that have accrued patients within 60 days of opening to enrollment have increased from 48.9 to 56.1%. A decline was noted in FY20, which we attribute to the COVID-19 pandemic. We have seen a decline in studies closed with no enrollment from 25.2 to 13.7%, notable progress toward our goal of 10% or fewer. Limiting these to industry-funded studies, we observed a significant decline from 36.0 to 23.8%.

Meeting enrollment expectations is critical to study success. We evaluated enrollment metrics across the enterprise as well as for interventional trials, specifically, as shown in Table 2. Total enrollment has increased since FY19. In FY20 and FY21, we observed a decrease in enrollment, coinciding with the pandemic, but have since seen enrollment rebound to pre-pandemic levels. Additionally, we have been able to further examine our enrollment data by capturing demographics of Race, Ethnicity, Sex, and Age to better inform Duke’s efforts to increase diversity in our research populations.

**Table 2.** Total enrollment numbers and demographics for fiscal years 2019–2022

	2019	2020	2021	2022
Total enrollment*	25,894	23,502	21,983	27,186
Enrollment in interventions**	12,935	9,612	9,443	14,799
**Demographics (%)**				
**Race**				
White	63%	63%	61%	63%
Black or African American	23%	22%	23%	23%
Asian	3%	3%	3%	3%
American Indian or Alaska Native	1%	1%	1%	1%
Native Hawaiian or Other Pacific Islander	0%	0%	0%	0%
2 or more races	2%	3%	2%	1%
Unknown	8%	9%	10%	9%
**Ethnicity**				
Non-Hispanic	87%	85%	85%	87%
Hispanic or Latino	5%	6%	6%	6%
Unknown	8%	9%	10%	7%
**Sex**				
Female	55%	57%	56%	58%
Male	45%	43%	44%	42%
**Age**				
Children/minors (under 18)	19%	20%	17%	14%
Young adults (18–34)	16%	17%	17%	23%
Middle-aged adults (35–64)	40%	41%	44%	34%
Older adults (65+)	27%	28%	27%	30%
Unknown	2%	2%	5%	2%

* Excludes studies using summary accrual in OnCore.

** Defined as an OnCore protocol type of treatment, screening, or prevention.

### Industry Study Start-up Timelines

Delays in study start-up are common yet costly and often have negative impacts throughout the study lifecycle [11,14]. To get better insight into our industry start-up performance, data collected through OnCore Task Lists were merged with data from iRIS and our contract tracking system to create a set of turnaround time metrics for each step in the industry study start-up process. These metrics were compared against a 90-day overall start-up target, as well as targets for each individual step (see Table 3). Beginning in FY21, all industry-funded budgets were built in OnCore, and Task Lists were used to track the budget and protocol calendar build processes. Turnaround times for the OnCore Calendar Creation step have been close to target. Each of the median days for the steps OnCore Calendar Finalized, SOM Finance Sign-off, and DOCR Calendar/Budget Signoff in FY21– FY23 has met or surpassed our targets (all central functions).

**Table 3.** Median calendar days per step in the study approval process for industry sponsored studies for fiscal years 2021 through Q2 of 2023

Step	Target number of days	FY 2021 *(N = 192)*	FY 2022 *(N = 245)*	FY 2023 (first half) *(N = 106)*
Step 1: Protocol Synced	0	0	0	0
Step 2: Draft Sponsor Budget Submitted	7	7	9	20
Step 3: Schedule Study Initiation Meeting	1	2	1	0
Step 4: Calendar Creation	7	8	12	11
Step 5: Study Initiation Meeting	7	7	6	6
Step 6: PI/CRC Calendar Approval	7	13	21	24
Step 7: Calendar Finalized	2	1	1	1
Step 8: CRU Budget Signoff	51	77	108	88
Step 9: SOM Finance	3	1	0	0
Step 10: DOCR Calendar / Budget Signoff	2	0	0	0
Parallel process only applicable to a subset of studies				
Step A1: Epic Clinical Build	14	14	21	4
Step A2: Epic Clinical Build Approved	3	17	33	17
Step A3: Epic Billing Build	3	4	6	7
Final step contingent on completion of all applicable steps above				
Step 11: Institutional Approval	3	8	16	19

In FY22, the data from OnCore Task Lists helped us pinpoint that time spent entering a budget by the study team into OnCore revealed a need for improvement. We addressed the issue by moving task responsibility to the central team. A small pilot of 40 studies evaluated a central budget input process and resulted in a 48% decrease (10 calendar days) for budget entry and a 17% decrease (37 calendar days) in the start-up timeline to Institutional Approval. This is reflected in the CRU Budget Signoff step in Table 3, where the FY22 median decreased from 108 calendar days to 88 calendar days in FY23.

This enhanced dataset helps identify bottlenecks in the workflow where further intervention is needed. Targeted opportunities exist to improve Draft Sponsor Budget Submitted, PI/CRC Calendar Approval, CRU Budget Signoff, and Epic Build Approval where timelines are increasing and are the responsibility of the study team. With this dataset, interventions can be prioritized based on impact to the overall process and allow for effective deployment of limited resources to optimize the study start-up process.

## Discussion

Implementing OnCore provided the opportunity to create standardized clinical research workflows for study start-up, conduct, and closeout and enhanced metric evaluation. These standard workflows allow for consistency, transparency, regulatory compliance, and identification of risks and bottlenecks. OnCore improved insight into our clinical research enterprise. In turn, this allows us to detect problems, identify improvement opportunities, and create strategies to impact performance. In addition, we are able to adapt to rapidly changing environmental conditions immediately.

### A Bolstered Clinical Research Infrastructure Allowed for Extreme Agility During the COVID-19 Pandemic

In response to the rapidly worsening COVID-19 outbreak, Duke issued a Shelter in Place order in March 2020. Using our established centralized infrastructure and systems, we quickly implemented a process to identify those studies that involved potentially lifesaving interventions that needed to remain open, and nonessential studies that could be rapidly spun down or paused. A rapid response to pivot for the COVID-19 pandemic would not have been as streamlined without OnCore and the complete and accurate real-time data view. We used OnCore to identify all active clinical research protocols and labeled each study with one of three tiers based on the level of impact that pausing the study could have on research participants’ health.

Centrally assigning a tier to all research studies in OnCore allowed for accurate ad hoc reporting on study visit activity, enrollment and identification of essential staff working on-site. We leveraged the tier structure and funding data in OnCore for a phased, comprehensive re-opening plan. Having an established central system to track tier designations, a central office to implement major changes efficiently, and a trained and organized workforce was fundamental to Duke’s ability to provide critical care to research patients during a very tumultuous time.

### Post Award OnCore Financials

Clinical research financial accountability and regulatory compliance are the responsibility of the CRU. Prior to OnCore, CRU finance teams used an Excel-based tool to track subject visits, milestone completion, invoicing, and payments. Collating institution-level financial reporting was labor-intensive, tedious, and subject to significant errors. Reporting was delayed by manual data collection and processing, creating a timing gap from when an issue emerged to recognition and the ability to address. Issues were far worse once recognized, resulting in a reactive problem-solving approach.

Using OnCore provides both detailed project level and overall financial status within a day of real time. From a post-award perspective, comprehensive documentation of detailed budgets, subject visit tracking, and the ability to use the modules to comply with contractual payment terms allows for accurate invoicing. The likelihood of missing costs or invoiceable items has been greatly reduced. CRUs have anecdotally acknowledged greater ability to capture and reconcile expenses and payments for studies. Investigating payment discrepancies is quicker and easier with OnCore’s added layer of transparency and accountability. As our post-award portfolio in OnCore expands, we are now able to track baseline and trend post-award data across the enterprise.

### Looking to the Future

By standardizing critical workflows, capturing detailed and accurate data and metrics, and providing users with direct access to real-time insight into research study performance, Duke is moving from an environment of endless fire fighting to one of proactive oversight and management. Based on the enhanced infrastructure, study start-up timeline dashboards for industry-funded clinical research protocols were recently launched. These dashboards use our enhanced start-up dataset to provide institutional, CRU, and individual protocol views of clinical research studies in the start-up phase, as well as metrics for studies that reached institutional approval. Protocol-level dashboards compare target versus actual days for each step of the start-up process. These dashboards provide real-time transparency and insights for CRU leaders and study staff to act upon.

The evolution of OnCore at Duke is iterative. We have continued engagement with the research community, taking feedback and input from users and communicating enhancement requests back to the vendor. Most recently, we requested the addition of gender identity fields to OnCore. This additional functionality will be incorporated into the next OnCore upgrade.

Informed clinical research is dependent on diverse enrollment, which is an area where more progress needs to be made [15–19]. Equity, Diversity, and Inclusion (EDI) is a strategic focus area for Duke with a particular focus on addressing equity in clinical research. To address this in clinical research, enrollment demographic dashboards are being created, leveraging the participant-level demographics in OnCore. These dashboards will allow Duke to better understand gaps in enrollment and representation and create strategies to ensure clinical research is advancing health care for all.

### Closing

Over the last 10 years, Duke clinical research has undergone an organizational transformation, creating a synergy between people, process and technology that has led to improvements in research quality, data transparency, and regulatory compliance. The revamping of DOCR created the standardized processes necessary to support and oversee such a large and complex portfolio of clinical research. Reforming the clinical research workforce enhanced staffing who implemented these standard processes through experience and knowledge. Finally, the implementation of OnCore provided the workflow management and data capture that allowed Duke to tie people, process, and technology to create a clinical research ecosystem that is collaborative and supports our cutting-edge clinician researchers. We believe this will optimize clinical research portfolio performance and ultimately, lead our efforts to change care.
